# Prevalence and determinants of persistent symptoms after treatment for Lyme borreliosis: study protocol for an observational, prospective cohort study (LymeProspect)

**DOI:** 10.1186/s12879-019-3949-8

**Published:** 2019-04-15

**Authors:** Hedwig D. Vrijmoeth, Jeanine Ursinus, Margriet G. Harms, Tizza P. Zomer, Stefanie A. Gauw, Anna D. Tulen, Kristin Kremer, Hein Sprong, Hans Knoop, Yolande M. Vermeeren, Barend van Kooten, Leo A. B. Joosten, Bart-Jan Kullberg, Joppe W. R. Hovius, Cees C. van den Wijngaard

**Affiliations:** 1 Department of Internal Medicine and Radboud Center for Infectious Diseases, Radboud University Medical Center, P.O. Box 9101, 6500 HB Nijmegen, the Netherlands; 2 Department of Internal Medicine, Division of Infectious Diseases & Center for Experimental and Molecular Medicine, Amsterdam UMC, University of Amsterdam, P.O. Box 22660, 1100 DD Amsterdam, the Netherlands; 3 National Institute for Public Health and Environment (RIVM), Center for Infectious Disease Control, P.O. Box 1, 3720 BA Bilthoven, the Netherlands; 4 Department of Medical Psychology, Amsterdam UMC, University of Amsterdam, P.O. Box 22660, 1100 DD Amsterdam, the Netherlands; 5 Lyme Center Apeldoorn, Gelre Hospital, P.O. Box 9014, 7300 DS Apeldoorn, the Netherlands

**Keywords:** Lyme disease, Borreliosis, Erythema Migrans, Borrelia, Persistent symptoms, Post-treatment Lyme disease syndrome, Study protocol

## Abstract

**Background:**

After antibiotic treatment of Lyme borreliosis, a subset of patients report persistent symptoms, also referred to as post-treatment Lyme disease syndrome. The reported prevalence of persistent symptoms varies considerably, and its pathophysiology is under debate. The LymeProspect study has been designed to investigate the prevalence, severity, and a wide range of hypotheses on the etiology of persistent symptoms among patients treated for Lyme borreliosis in the Netherlands.

**Methods:**

LymeProspect is a prospective, observational cohort study among adults with proven or probable Lyme borreliosis, either erythema migrans or disseminated manifestations, included at the start of antibiotic treatment. During one year of follow-up, participants are subjected to questionnaires every three months and blood is collected repeatedly during the first three months. The primary outcome is the prevalence of persistent symptoms after treatment, assessed by questionnaires online focusing on fatigue (CIS, subscale fatigue severity), pain (SF-36, subscale pain) and neurocognitive dysfunction (CFQ). Potential microbiological, immunological, genetic, epidemiological and cognitive-behavioral determinants for persistent symptoms are secondary outcome measures. Control cohorts include patients with long-lasting symptoms and unconfirmed Lyme disease, population controls, and subjects having reported a tick bite not followed by Lyme borreliosis.

**Discussion:**

This article describes the background and design of the LymeProspect study protocol. This study is characterized by a prospective, explorative and multifaceted design. The results of this study will provide insights into the prevalence and determinants of persistent symptoms after treatment for Lyme borreliosis, and may provide a rationale for preventive and treatment recommendations.

**Trial registration:**

NTR4998 (Netherlands Trial Register). Date of registration: 13 February 2015.

**Electronic supplementary material:**

The online version of this article (10.1186/s12879-019-3949-8) contains supplementary material, which is available to authorized users.

## Background

Lyme borreliosis is caused by tick-borne *Borrelia burgdorferi* sensu lato genospecies. The incidence of tick bites and cases of Lyme borreliosis has increased substantially over the past decades. In the Netherlands, annually over one million people report a tick bite, and more than 25.000 cases of Lyme borreliosis are diagnosed [1–3]. The most common manifestation of Lyme borreliosis in Europe is erythema migrans, a red or bluish-red expanding skin lesion arising within days to weeks at the site of the tick bite. A minority of infected individuals develops disseminated Lyme borreliosis, of which Lyme neuroborreliosis, Lyme arthritis and acrodermatitis chronic atrophicans (ACA) are most common [4].

Although Lyme borreliosis generally responds well to antibiotic therapy, some patients report persistent symptoms after treatment. However, the prevalence of persistent symptoms in literature varies considerably, between 0 and 48% [2, 5–17], and many of these studies lack proper controls. Case definition, Lyme borreliosis manifestation, follow-up, geographic location, and delay between onset of symptoms and treatment might explain the divergence in prevalence. Whereas the initial objective manifestation usually resolve, disabling symptoms such as fatigue, musculoskeletal pain and neurocognitive disturbances can last for months or even years, greatly affecting quality of life. When present for more than six months, post-treatment Lyme Disease Syndrome (PTLDS) is the term frequently used to describe these persistent symptoms [9, 18, 19].

The prevalence as well as the underlying pathology of persistent symptoms after treatment for Lyme borreliosis are not clearly defined and under debate. Consequently, uniform guidelines on prevention and treatment of persistent symptoms are lacking. These uncertainties have led to great societal concern and dissatisfaction among patients. A broad range of hypotheses exists regarding the pathophysiology underlying persistent symptoms, ranging from microbiological, immunological, genetic, cognitive-behavioral, clinical, to epidemiological explanations. The LymeProspect study has been designed to test this broad range of hypotheses on persistent symptoms in a large cohort of patients with confirmed Lyme borreliosis. In this prospective, observational study, patients are followed from the start of antibiotic treatment during one year. This design allows us to assess the prevalence of persistent symptoms after treatment for Lyme borreliosis and to identify determinants of these symptoms. In this paper, we describe the study protocol in detail.

## Methods

### Study design

A multi-center prospective, observational cohort study with one year of follow-up is performed to determine the prevalence and severity of persistent symptoms after antimicrobial treatment for active Lyme borreliosis, and to assess determinants for development of these symptoms. Potential microbiological, immunological, genetic, clinical, cognitive-behavioral, and epidemiological determinants are assessed in patients with confirmed early or disseminated Lyme borreliosis. This study is a collaboration between the Dutch National Institute for Public Health and the Environment (RIVM, Bilthoven, the Netherlands) and the clinical expert centers for Lyme borreliosis at Amsterdam UMC (location AMC, University of Amsterdam, Amsterdam, the Netherlands), and Radboud university medical center (Radboudumc, Nijmegen, the Netherlands), in collaboration with the Lyme center Apeldoorn (Gelre Hospital, Apeldoorn, the Netherlands). The study has been approved by the medical ethics committee (METC) Noord-Holland (NL50227.094.14), and is conducted according to the principles of the Declaration of Helsinki.

### Study population

This prospective study comprises adults with probable or proven Lyme borreliosis - either local infection or disseminated disease - in the Netherlands. Recruitment has started in April 2015. Recruitment of children has started in 2017 and results will be reported separately. Case definitions include clinical and laboratory criteria, and are largely based on the case definitions established by ESGBOR (Additional file 1: Table S1) [4]. Patients are included within 7 days after, but preferably before, initiation of antibiotic treatment. Patients with signs and symptoms attributed to a previous episode of Lyme borreliosis are excluded (Table 1). A separate cohort of patients with long-lasting symptoms attributed to Lyme borreliosis, without clinical and microbiological confirmation, is enrolled in the unconfirmed Lyme borreliosis control group. Inclusion criteria in this control group include symptoms (e.g. fatigue, cognitive complaints, myalgias and arthralgias) for more than 6 months with a severity above the Dutch norm score, as assessed by a combined questionnaire based on the CIS, SF-36 and CFQ as described below; negative serology (*B. burgdorferi* s.l. IgG ELISA or a combined IgM/IgG ELISA); and attribution of symptoms to Lyme borreliosis either because symptoms originated within one month after a documented tick bite, or because of a positive result of a non-recommended test (e.g., LTT or other commercially available cellular test, CD57 analysis) (Table 1). In addition, two other control cohorts are available for comparison. Firstly, a population control cohort consists of subjects randomly recruited nationwide, representing the general population. Data from patients and from these controls are matched by age, gender, geographical region, and month of enrollment. Secondly, a tick bite control cohort includes subjects who have reported a tick bite without having developed clinical evidence for a *Borrelia* infection at baseline or during follow-up. The same questionnaires are administered to individuals in all control cohorts and the prospective study patients during one year of follow-up.

**Table 1:** Inclusion and exclusion criteria

Patients with confirmed Lyme borreliosis	
Inclusion criteria:	
• Patients ≥18 years with confirmed proven or probable early localized or disseminated Lyme borreliosis manifestation as specified in Additional file 1: Table S1;	
• In case of an EM reported at www.tekenradar.nl, the EM has been present < 3 months and the clinical diagnosis has been confirmed by the general practitioner (criteria for clinical diagnosis are described in Additional file 1: Table S1);	
• Subjects live or stay on the mainland of the Netherlands.	
Exclusion criteria:	
• Subjects unable to provide informed consent or not having a sufficient command of the Dutch language;	
• Subjects who started antibiotic treatment > 4 days before inclusion (for subjects included through the website www.tekenradar.nl) or > 7 days before inclusion (for subjects included through the clinical expert centers for Lyme borreliosis);	
• Subjects who have ongoing signs or symptoms attributed to a previous episode of Lyme borreliosis.	
Patients with unconfirmed Lyme borreliosis	
Inclusion criteria:	
• Myalgia, arthralgia, neuralgia, concentration disorders, cognitive disturbances, with or without fatigue, present for ≥6 months at baseline	
• Severity of symptoms assessed by the CIS, SF-36 and/or CFQ questionnaire above the Dutch norm scores;	
• Subjects have a negative serological test for *B. burgdorferi s.l.* (IgG ELISA or C6 IgM/IgG ELISA), but have a history of an unconfirmed suspicion of Lyme disease, based on	
• a positive result for a non-recommended diagnostic test (e.g., cellular tests, CD57 analysis, viable blood analysis, bioresonance), or	
• onset of disease symptoms that have started within one month after a documented tick bite.	
Exclusion criteria:	
• Subjects are unable to give informed consent or do not have sufficient command of the Dutch language.	

### Recruitment, inclusion and follow-up of participants

Recruitment, inclusion and follow-up of participants occur both online and at the clinical expert centers for Lyme borreliosis. The national website www.tekenradar.nl offers secure eligibility screening, inclusion and follow-up of participants with confirmed Lyme borreliosis after either self-registration or registration by a medical doctor (GP or specialist). After online enrollment, the participant’s medical doctor is contacted to confirm the diagnosis. Written informed consent is obtained from eligible patients. After inclusion, blood collection tubes are delivered at the patient’s home address by courier service, and blood is collected at a local blood draw service at baseline and after 6 weeks. Blood samples are sent overnight to the study laboratories and processed immediately upon arrival, to ensure the processing is completed within 24 h after blood collection.

In addition to online enrollment, study subjects as well as unconfirmed Lyme borreliosis controls are enrolled at the participating clinical expert centers. For these patients, blood samples are collected and processed immediately after inclusion. From a subset of patients with skin manifestations, skin biopsies are obtained after additional informed consent.

### Measurements

For patients with confirmed Lyme borreliosis, standard demographical characteristics are reported at baseline, including gender, age, and highest educational level. Comorbidities are reported at baseline, and new medical diagnoses are evaluated during follow-up. Details on exposure to ticks, previous Lyme borreliosis episodes and the current Lyme borreliosis manifestation and treatment are collected. Patients with erythema migrans or other skin manifestations, who are included online are requested to upload a photograph of their skin lesion, for blinded evaluation by independent experts. Signs and symptoms are assessed at baseline and after 3, 6, 9 and 12 months, by online questionnaires on fatigue, pain and neurocognitive dysfunction (Table 2). Fatigue severity is assessed by the fatigue severity subscale of the Checklist Individual Strength (CIS) [20]. Scores range from 8 to 56, and scores of 35 or higher reflect severe fatigue. The Medical Outcomes Survey Short Form-36 (SF-36) pain subscale is used to assess severity and impact of pain. Significant impairment due to pain is reflected by a score of 55 or lower, based on Dutch norm scores [21]. Neurocognitive functioning is assessed with the Dutch version of the Cognitive Failure Questionnaire (CFQ) [22]. Clinically significant complaints on neurocognitive functioning are reflected by a score of 44 or higher. Clinical and cognitive-behavioral parameters are assessed by online questionnaires as well, including somatic symptoms (PHQ-15) [23], physical and social functioning (SF-36, subscales physical functioning and social functioning) [24], health care use, absenteeism from work, co-morbidity (adapted from TiC-P) [25], pre-existent symptoms (adapted from PREDIS) [26], illness perception (Brief IPQ) [27], cognitive and behavioral responses to symptoms (CBRSQ) [28], psychological distress (HADS) [29], self-efficacy with respect to pain and fatigue (SES, PCS) [30, 31], and the level of physical activity (IPAQ) [32] (Table 2). Blood is collected at several time points for microbiological, immunological and genetic analyses (Table 2). For detection of *B. burgdorferi* s.l.-specific antibodies, a total Ig C6 ELISA (Immunetics, Boston, MA, USA) is performed on all samples, followed by IgM and IgG immunoblot analysis (Mikrogen GmbH, Neuried, Germany) for confirmation of C6 ELISA positive or borderline results. Furthermore, serology for other tick-borne pathogens is performed on at least all samples collected at six weeks. Immunofluorescence analysis (Focus Diagnostics, Cypress, CA, USA) is used for the detection of antibodies directed to *Babesia* spp., *Rickettsia* spp. and *Anaplasma phagocytophilum*. An IgM and IgG ELISA (TestLine Clinical Diagnostics, Brno, Czech Republic) for *tick borne encephalitis* (TBE) virus is conducted for patients with persistent symptoms and matched patients without persistent symptoms. Antibodies against *B. miyamotoi* are assessed by an experimental assay [33]. All blood samples are analyzed with (multiplex) real-time PCRs, based on various genes specific for *B. burgdorferi* s.l., *B. miyamotoi*, *A. phagocytophilum*, *Candidatus Neoehrlichia mikurensis*, spotted fever *Rickettsia’s*, *Bartonella* spp., and a wide range of *Babesia* spp.. In patients with cutaneous Lyme borreliosis manifestations, skin biopsies are collected at baseline after additional consent, and assessed for *B. burgdorferi* s.l. culture and molecular detection of *B. burgdorferi* s.l. by PCR. Multilocus sequence typing (MLST) analysis is performed to type clinical *B*. *burgdorferi* s.l. isolates from skin biopsies.

**Table 2:** Data collection and measurements for all patients with confirmed Lyme borreliosis

	Baseline	10 days	2 weeks	6 weeks	3 months	6 months	9 months	12 months
Written information and informed consent	X							
Baseline characteristics	X							
Physical examination	X^a^				X^ac^	X^ac^	X^ac^	X^ac^
Recording Lyme manifestation, treatment and concomitant medication	X		X^b^		X	X	X	X
Recording adverse events	X		X^b^		X	X		X^a^
*Questionnaires*								
Primary outcome: CIS (subscale fatigue), SF-36 (subscale pain), CFQ	X				X^1^	X	X^1^	X
Clinical parameters: PHQ-15, SF-36 (subscale physical functioning and subscale social functioning), TiC-P (health-care use and absenteeism of work)	X				X	X	X	X
Cognitive-behavioral parameters: brief IPQ, CBRSQ, HADS, SES-F, PCS, IPAQ	X				X	X		
Comorbidities: TiC-P (co-morbidity list)	X							X
Comorbidities: PREDIS	X							
*Laboratory measurements*								
*B. burgdorferi* s.l. serology	X	X^ac^		X	X^ac^			
Serology other TBPs^3^	X^2^			X^2^				
PCR *B. burgdorferi* s.l. and other TBPs^3^ (blood)	X			X				
Genome wide association studies	X^4^							
Cytokine measurements in cell culture supernatants	X			X				
Gene expression micro-arrays on ex vivo stimulated PBMCs	X^5^			X^5^				
Skin biopsies: culture, MLST, PCR *B. burgdorferi* s.l. and other TBPs, gene-expression profiling	X^6^							

^a^Patients included through the clinical expert centers for Lyme borreliosis only

^b^Patients included through the website www.tekenradar.nl only

^c^These visits and laboratory measurements can be left out if patients are not able or not willing to. This is regarded as an allowed deviation from the protocol

^1^CIS questionnaire only short version, to limit the burden for patients

^2^In cases (patients with persistent symptoms) and twice as much controls (patients without persistent symptoms), starting with the 6 weeks sample. If borderline or positive, the baseline sample will be tested as well

^3^Serology on *Babesia* spp., *Rickettsia conorii*, *Anaplasma* phagocytophilum (commercially available) and *Borrelia miyamotoi* (experimental). Quantitative PCR including *Babesia* spp., *Rickettsia* spp., *Anaplasma* phagocytophilum, *Neoehrlichia mikurensis*, as well as a pan relapsing fever *Borrelia* qPCR (if positive, a specific *Borrelia miyamotoi* qPCR will be performed)

^4^In the first consecutive included 600 patients with erythema migrans

^5^In a selection of patients, included through the clinical expert centers for Lyme borreliosis

^6^In patients with a skin manifestation included through the clinical expert centers for Lyme borreliosis (only after additional consent). Both the affected and contra-lateral side will be investigated

*Abbreviations:* (q)*PCR* (quantitative) polymerase chain reaction, *TBPs* tick-borne pathogens, *PBMCs* peripheral blood mononuclear cells, *MLST* Multilocus sequence typing. For the abbreviations of the various questionnaires, see the main text

Immunological assays include measurements of pro- and anti-inflammatory cytokines in cell culture supernatants of ex vivo stimulated whole blood and PBMCs, stimulated with RPMI (medium control), viable *B. burgdorferi* s.l. (mix of *B. burgdorferi* s.s., *B. garinii* and *B. afzelii)* at different multiplicities of infection (MOI)*,* lipopolysaccharide (LPS), Pam3cys, and heat killed *Candida albicans* blastoconidia. Interleukin (IL)-1ß, IL-6, IL-1Ra and IL-10 produced after 24 h of exposure to these stimuli are measured with commercial ELISA kits. In a subset of patients, also data from pre-market and commercial cellular tests will be available. In addition, genome wide association studies (GWAS) are performed on DNA extracts from EDTA blood from the first consecutive 600 patients included with erythema migrans, to identify associations between single-nucleotide polymorphisms (SNPs) and the development of persistent symptoms, ex-vivo cytokine production profiles, or other determinants assessed in this study. Finally, in a selected subset of patients, gene expression profiling (microarray) is performed on skin biopsies from the lesion and contralateral healthy skin as well as on stimulated PBMCs.

From subjects in the unconfirmed Lyme borreliosis control group, blood is collected at baseline only. These controls are subjected to the same measurements as the confirmed patients, with the exception of genetic analysis and skin biopsies, whereas for the population control group and the tick bite control group only questionnaire data are obtained.

### Outcome measures and data analysis

The primary outcome measure is the prevalence and severity of persistent symptoms in patients with confirmed Lyme borreliosis after treatment, assessed by standardized questionnaires on fatigue, pain and neurocognitive functioning. Persistent symptoms are defined as an impaired score for fatigue severity (CIS, subscale fatigue, score 35 or higher), pain (SF-36, subscale pain, score 55 or lower), or impaired neurocognitive functioning (CFQ, score 44 or higher), started within six months after treatment for confirmed Lyme borreliosis, and lasting for at least 6 months, as assessed by questionnaires administered at baseline, and 3, 6, 9 and 12 months after inclusion. Patients with scores exceeding the cutoff scores for fatigue, pain or neurocognitive functioning during at least six months are categorized as cases, i.e. Lyme borreliosis patients with persistent symptoms. To correct for baseline symptoms unrelated to Lyme borreliosis, the prevalence of symptoms in the population control cohort and tick bite control cohort are used. Variability and seasonality in scores on several questionnaires obtained during one year are compared between patients with confirmed Lyme borreliosis, the population control group and the tick bite control group. A separate analysis is performed on patients with persistent symptoms lasting less than 6 months.

Secondary outcome measures are potential microbiological, immunological, genetic, clinical, cognitive-behavioral, and epidemiological determinants of persistent symptoms. The association of these parameters with development of persistent symptoms is assessed by comparing results of confirmed Lyme borreliosis patients with persistent symptoms (cases) and without persistent symptoms (controls). Prediction rules for the risk and severity of persistent symptoms for individual patients will be developed. Determinants for persistent symptoms identified in patients with confirmed Lyme borreliosis are also assessed in the unconfirmed Lyme borreliosis control group in an exploratory manner.

### Sample size

As mentioned above, the reported percentage of patients developing persistent symptoms after treatment for Lyme borreliosis varies widely from 0 to 48%. For the present study, a prevalence of 5% is assumed, based on a retrospectively estimated annual incidence rate of persistent symptoms in patients with erythema migrans and disseminated Lyme borreliosis in the Netherlands [2]. At a 5% prevalence of persistent symptoms for a cohort of 2000 patients, a 25% lower proportion for a determinant can be detected in patients without symptoms, if present in at least 57% of patients with persistent symptoms (power 80%, alpha 5%). In a cohort of 1500 patients, a 25% lower proportion can be detected for a determinant that is present in at least 66% of cases with persistence of symptoms, and for a cohort of 1000 patients for one that is present in at least 77% of cases (power 80%, alpha of 5%). When the prevalence of persistent symptoms would be higher, the power to detect these differences increases.

## Discussion

The LymeProspect study evaluates the prevalence and severity of persistent symptoms after treatment for Lyme borreliosis, and aims to identify determinants for these symptoms. This study is the first to apply a prospective design, investigating a broad range of hypotheses on the etiology of persistent symptoms in a large cohort of patients with confirmed Lyme borreliosis. Previous studies have described the prevalence of persistent symptoms attributed to Lyme borreliosis in the United States and in Europe. Several studies have reported associations between individual microbiological, immunological, genetic, clinical and cognitive-behavioral factors and persistent symptoms after treatment in specific groups of patients. Firstly, persistence of *Borrelia* infection after antibiotic treatment has been suggested as a cause of persistent symptoms after treatment [34], although this is difficult to detect with the current diagnostic tools, and randomized trials have not found beneficial effects of prolonged antibiotic treatment in patients with persistent symptoms [35–38]. Other microbiological hypotheses include culture positivity, infection by specific *Borrelia burgdorferi* s.l. genospecies or strains, and co-infection with other tick-borne pathogens [39–42], although there is no convincing evidence of long-term infection with other tick-borne pathogens in patients with persistent symptoms attributed to Lyme disease [43]. Secondly, an ongoing aberrant immune response and underlying genetic polymorphisms may play a role. This has been specifically well-studied in patients with antibiotic-refractory Lyme arthritis [44–46]. Furthermore, several cytokines or cytokine profiles have been related to persistence of symptoms after treatment for Lyme borreliosis [15, 47–50]. Thirdly, in a cognitive-behavioral model, the persistence of symptoms was related to beliefs and behavior of the patients in response to these symptoms. This model has been successfully applied to persistent symptoms in other conditions [51, 52], and previous studies have suggested that psychological factors could play a role in functional outcome in patients with Lyme borreliosis [10, 53]. Finally, clinical and epidemiological factors could be associated with persistence of symptoms, including age, clinical signs, co-morbidity, Lyme borreliosis manifestation, duration of symptoms before start of treatment, type of antibiotic treatment, and prior exposure to tick bites or *Borrelia* infection [14, 54].

However, conclusions of previous findings vary widely and are difficult to be generalized due to heterogeneity in case definitions, antibiotic treatment regimes, follow-up periods, control groups and primary outcomes. The strengths of the current study are its size, prospective approach, and explorative and multifaceted design, enabling a thorough analysis of the prevalence and determinants of persistent symptoms of Lyme borreliosis in the Netherlands. The use of validated questionnaires with norm scores and the availability of additional population and tick bite control groups will allow to accurately compare the prevalence and severity of symptoms in the patient cohort to the background prevalence. The control groups will also allow correction for development of non-specific symptoms over time and their seasonal changes.

The majority of participants are expected to be diagnosed with erythema migrans and to be enrolled online. To ascertain that subjects meet the inclusion criteria, medical information about the diagnosis is obtained from their physician in a standardized fashion, as well as a photograph of the skin lesions, which will be evaluated by independent experts. Serological evaluation at two time points could further contribute to the confirmation of active Lyme borreliosis. For patients who are enrolled through the clinical Lyme expertise centers and have consented to skin biopsy, *B. burgdorferi* s.l. culture and qPCR on skin biopsies for *B. burgdorferi* s.l. will enable further laboratory confirmation of the diagnosis.

Patients are encouraged to provide baseline blood samples before initiation of antibiotic therapy. To assess a potential effect of antibiotic treatment on microbiological and immunological endpoints, duration of prior treatment will be explored in sensitivity analyses. We have ruled out a significant effect of overnight shipping of blood samples on immunological outcomes in prior pilot studies (unpublished data).

The primary outcome measure has been defined as the presence of fatigue (CIS), pain (SF-36) and/or neurocognitive complaints (CFQ) presenting within six months after initial diagnosis and treatment, and lasting for at least six months. Fatigue, pain and neurocognitive complaints have been reported as major symptoms reported after Lyme borreliosis, and constitute the definition of PTLDS. Repeated assessment of symptoms every three months during one year of follow-up will enable to assess the time course of symptoms, and to detect disabling symptoms lasting shorter than six months as well. Furthermore, the impact of symptoms on general functioning, as measured by physical and social functioning, health-care use and absenteeism at work, is assessed. Subjects experiencing symptoms, or those preoccupied with Lyme borreliosis, could be more inclined to fill in questionnaires at baseline and to complete the follow-up, possibly leading to selection bias. This underscores the importance of the comparison of our primary outcomes in the patient cohort with the various control groups, since all three groups have the same scheme of follow-up and risk of bias.

In conclusion, the LymeProspect study is expected to provide additional insights into the prevalence and severity of persistent symptoms after antibiotic treatment for Lyme borreliosis. In addition, the study will identify determinants for persistent symptoms. These findings may lead to the development of prediction models for individual patients and may guide future research on preventive and therapeutic strategies of persistent symptoms after treatment for Lyme borreliosis.

## Additional file

Additional file 1: **Table S1**. Clinical and laboratory criteria for inclusion of patients with confirmed Lyme borreliosis. Clinical and laboratory inclusion criteria for patients with confirmed Lyme borreliosis are described in detail. Criteria are largely based on case definitions published by Stanek et al. and alternative causes for symptoms should be excluded by the primary caregiver ^4^. *The CXCL-13 cut-off value is laboratory dependent. ^#^As determined by neurological assessment or electromyogram. ^^^Preferably through synovial fluid puncture or synovium biopsy. ^%^After a cardiologist has been consulted. (DOCX 17 kb)

## Abbreviations

(q)PCR: (quantitative) polymerase chain reaction
ACA: Acrodermatitis chronic atrophicans
CBRSQ: Cognitive Behavioural Responses to Symptoms Questionnaire
CFQ: Cognitive failure questionnaire
CIS: Checklist Individual Strength
CSF: Cerebrospinal fluid
DNA: Desoxyribo Nucleic Acid
EDTA: Ethylenediaminetetraacetic acid
ELISA: Enzyme-Linked Immuno Sorbent Assay
EM: Erythema migrans
GP: General practitioner
GWAS: Genome wide association studies
HADS: Hospital Anxiety and Depression Scale
IFA: Immunofluorescence assay
IL: Interleukine
IPAQ: International Physical Activity Questionnaire
IPQ: Illness perception questionnaire
LPS: Lipopolysaccharide
LTT: Lymphocyte transformation test
MLST: Multilocus sequence typing
MOI: Multiplicity of infection
PBMC: Peripheral blood mononuclear cell
PBMCs: Peripheral blood mononuclear cells
PCS: Pain Catastrophizing Scale
PHQ: Patient Health Questionnaire
PTLDS: Post-treatment Lyme disease syndrome
s.l.: sensu lato
SES: Self-Efficacy Scale
SF-36: SF-36 Health Status Inventory
SNP: Single nucleotide polymorphism
spp.: species
TBE: Tick borne encephalitis
TBP: Tick-borne pathogens
Tic-P: Trimbos/iMTA questionnaire for Costs associated with Psychiatric Illness
