# Supplementary material for: Prevalence and determinants of persistent symptoms after treatment for Lyme borreliosis: study protocol for an observational, prospective cohort study (LymeProspect)
Source: BMC Infect Dis. 2019 Apr 15;19:324. doi: 10.1186/s12879-019-3949-8 (PMC6466793; doi:10.1186/s12879-019-3949-8)
Supplement: Supplementary file 1 — Table S1. Clinical and laboratory criteria for inclusion of patients with confirmed Lyme borreliosis. Clinical and laboratory inclusion criteria for patients with confirmed Lyme borreliosis are described in detail. Criteria are largely based on case definitions published by Stanek et al. and alternative causes for symptoms should be excluded by the primary caregiver 4. *The CXCL-13 cut-off value is laboratory dependent. #As determined by neurological assessment or electromyogram. ^Preferably through synovial fluid puncture or synovium biopsy. %After a cardiologist has been consulted. (DOCX 17 kb) [file 12879_2019_3949_MOESM1_ESM.docx]

| Definitions of serological results:  - Early infection (symptoms <8 weeks):   - Positive *B. burgdorferi* s.l. IgM EIA/ELISA with positive *B. burgdorferi* s.l. IgM Immunoblot and negative *B. burgdorferi* s.l*.* IgG Immunoblot.   *or*   - Positive *B. burgdorferi* s.l. IgM/IgG EIA/ELISA with positive *B. burgdorferi* s.l*.* IgM Immunoblot and negative *B. burgdorferi* s.l. IgG Immunoblot.   *and/or*   - Seroconversion from negative or borderline IgG *B. burgdorferi* s.l. EIA/ELISA to positive IgG *B. burgdorferi* s.l. EIA/ELISA with positive IgG *B. burgdorferi* s.l. immunoblot.   *or*   - Seroconversion from negative or borderline IgM/IgG *B. burgdorferi* s.l*.* EIA/ELISA to positive IgM/IgG *B. burgdorferi* s.l. EIA/ELISA with positive IgG *B. burgdorferi* s.l. immunoblot.   - Late infection (symptoms >8 weeks):   - Positive *B. burgdorferi* s.l. IgG EIA/ELISA with positive *B. burgdorferi* s.l. IgG Immunoblot.   *and/or*   - Positive *B. burgdorferi* s.l. IgM/IgG EIA/ELISA with positive *B. burgdorferi* s.l. IgG Immunoblot. | | |
| --- | --- | --- |
| **Lyme manifestation** | **Clinical criteria** | **Laboratory criteria** |
| ***Early (localized) Lyme disease*** | | |
| Typical erythema migrans | Centrifugally expanding red or red-bluish macule or ring > 5 cm, without vesicles, papulae, desquamation or infiltration, regardless the observation of a tick bite. | None |
| Atypical erythema migrans with observed tick bite | Centrifugally expanding red or bluish-red macule or ring >5 cm, with vesicles, papulae, desquamation or infiltration. | None |
| Atypical erythema migrans, without observed tick bite | Centrifugally expanding red or bluish-red macule or ring >5 cm, with vesicles, papulae, desquamation or infiltration. | 1. Serological profile matching early infection   *and/or*   1. Positive *B. burgdorferi* s.l. PCR on skin biopsy   *and/or*   1. Positive *B. burgdorferi* s.l. culture on skin biopsy |
| Early (sub)acute symptoms without erythema migrans | Fever (>38.3 ̊C) or subfebrile temperature (37.8 ̊C-38.3 ̊C) AND myalgia or arthralgia. Symptoms are present for less than 3 months, and started within 1 month after a documented tick bite. | Serological profile matching early infection |
| ***Borrelial lymphocytoma*** | | |
| Proven *Borrelial* lymphocytoma | Painless smooth bluish-red nodule or plaque with a diameter of at least 1 cm, usually found on the ear lobe or helix, nipple or scrotum. | 1. Positive *B. burgdorferi* s.l. PCR or culture on skin biopsy and/or positive *B. burgdorferi* s.l. PCR or culture on blood if performed in a participating laboratory   *and/or*   1. Histopathology showing polyclonal B-lymphocytes infiltration, with positive spirochete staining   *and/or*   1. Histopathology showing polyclonal B-lymphocytes infiltration, without positive spirochete staining with a serological profile matching early infection |
| Probable *Borrelial* lymphocytoma | Painless smooth bluish-red nodule or plaque with a diameter of at least 1 cm, usually found on the ear lobe or helix, nipple or scrotum. | 1. Histopathology showing polyclonal B-lymphocytes infiltration, without positive spirochete staining   *or*   1. A serological profile matching early infection |
| ***Multiple erythema migrans*** | | |
| Proven multiple erythema migrans | Multiple red or blue-red skin lesions, oval or round shaped. | 1. Positive *B. burgdorferi* s.l. PCR of culture on skin biopsy   *and/or*   1. Positive *B. burgdorferi* s.l. PCR or culture on blood (if performed in a participating laboratory) |
| Probable multiple erythema migrans | Multiple red or blue-red skin lesions, oval or round shaped. The lesions are homogeneous and have a sharp border. | A serological profile matching early or late infection |
| ***Lyme neuroborreliosis*** | | |
| Proven Lyme neuroborreliosis | Meningo‐(poly)radiculoneuritis, meningitis,  myelitis, encephalitis, cerebral vasculitis  (presenting as a cerebrovascular accident), unilateral or bilateral facial palsy, or involvement of other cranial nerves. | 1. Pleocytosis and intrathecal specific *B. burgdorferi* s.l. antibody formation   *and/or*   1. Pleocytosis and elevated CXCL13 in cerebrospinal fluid (CSF)^*^   *and/or*   1. Positive PCR for *B. burgdorferi* s.l. or culture for *B. burgdorferi* s.l. on CSF |
| Probable Lyme neuroborreliosis | Meningo‐(poly)radiculoneuritis, meningitis,  myelitis, encephalitis, cerebral vasculitis  (presenting as a cerebrovascular accident), unilateral or bilateral facial palsy, or involvement of other cranial nerves. | 1. Pleocytosis and a serological profile matching early infection   *or*   1. Intrathecal specific *B. burgdorferi* s.l. antibody formation and a serological profile matching early or late infection   *or*   1. Elevated CXCL13 in CSF^*^ and a serological profile matching early or late infection |
| Probable Lyme polyneuropathy | Objective polyneuropathy^#^ together with skin lesions compatible with an acrodermatitis chronic atrophicans (see criteria ACA). | A serological profile matching late infection |
| ***Lyme arthritis*** | | |
| Proven Lyme arthritis | Persistent or recurrent swelling of one or more joints (synovitis), mostly the knee. | A positive PCR or culture of *B. burgdorferi* s.l. on synovial fluid or synovium |
| Probable Lyme arthritis | Persistent or recurrent swelling of one or more joints (synovitis), mostly the knee. | 1. A serological profile matching late infection   *and*   1. another causative explanation for the arthritis has been excluded after consulation by a rheumatologist^^^ |
| ***Acrodermatitis chronic atrophicans (ACA)*** | | |
| Proven ACA | Red or bluish-red discoloration of the skin with limited swelling end/or atrophy. | A positive PCR or culture of *B. burgdorferi* s.l. on skin biopsy |
| Probable ACA | Red or bluish-red discoloration of the skin with limited swelling end/or atrophy. | A serological profile matching late infection |
| ***Other, less frequent disseminated Lyme disease manifestations*** | | |
| Probable Lyme carditis | New onset of atrioventricular conduction disorder (first, second or third degree), or new onset of clinical symptoms of a peri-myocarditis together with at least one symptom of an early or late disseminated Lyme borreliosis <6 weeks before cardiac symptoms started. | 1. A serological profile matching late infection   *and*   1. another causative explanation for the carditis has been excluded^%^ |
| Proven Lyme uveitis | Uveitis (anterior, intermedia, posterior, or panuveitis) | A positive PCR *B. burgdorferi* s.l. or culture on vitreous fluid |
| Probable Lyme uveitis | Uveitis (anterior, intermedia, posterior, or panuveitis) | A serological profile matching early infection |
